# Differences in Vaginal Microbiota Composition Between Infertile and Fertile Patients: A Prospective Study

**DOI:** 10.3390/diagnostics15192544

**Published:** 2025-10-09

**Authors:** Pei-Chen Chen, Shih-Fen Chen, Wei-Tung Hung, Yu-Ying Lin, Ling-Chun Lin, Jen-Hung Wang, Pao-Chu Chen

**Affiliations:** 1 Department of Obstetrics and Gynecology, Hualien Tzu Chi Hospital, Buddhist Tzu Chi Medical Foundation, Hualien 97004, Taiwan; 2 Master and Ph.D. Programs in Pharmacology and Toxicology, School of Medicine, Tzu Chi University, Hualien 97004, Taiwan; 3 Reproductive Health and IVF Center, Hualien Tzu Chi Hospital, Buddhist Tzu Chi Medical Foundation, Hualien 97004, Taiwan; 4 Institute of Medical Sciences, Tzu Chi University, Hualien 97004, Taiwan; aphrodite@gms.tcu.edu.tw; 5 Master Program in Biomedical Sciences, School of Medicine, Tzu Chi University, Hualien 97004, Taiwan; 6 Center for Clinical Epidemiology and Biostatistics, Hualien Tzu Chi Hospital, Buddhist Tzu Chi Medical Foundation, Hualien 97004, Taiwan; paulwang@tzuchi.com.tw

**Keywords:** infertility, lactobacillus, vaginal microbiota, 16S rRNA gene

## Abstract

**Background/Objectives**: Dysbiosis of the vaginal microbiota, particularly the loss of Lactobacillus spp. dominance, is linked to female infertility. While community state types (CSTs) I–III and V have been studied extensively, CST IV remains underexplored. The aim of this prospective study was to compare vaginal microbiota composition—specifically CST IVA and IVB—between fertile and infertile women. **Methods**: Vaginal samples were collected from 22 women (15 infertile, 7 fertile) using cervical brushes and analyzed via 16S rRNA gene sequencing. DNA was extracted, and V3–V4 regions were sequenced using the Illumina MiSeq platform. Taxonomic classification was performed with QIIME 2 and the Greengenes database. Differences in microbial composition were assessed using the Wilcoxon rank-sum test (*p* < 0.05) in SPSS v21.0. **Results**: Infertile women showed lower relative abundances of *Lactobacillus* spp. (31.54% vs. 42.32%) and *Oscillospira* spp. relative to fertile women. CST IV was more frequent in the infertile group (29.75% vs. 21.61%). Within CST IV, CST IVA accounted for a higher proportion in infertile women (7.0% vs. 0.94%), with *Prevotella* spp. representing 95.18% of CST IVA in infertile subjects, as opposed to the figure of 69.77% in fertile counterparts. No clear differences in CST IVB were observed between groups. **Conclusions**: Increased prevalence of *Prevotella* spp. in CST IVA may contribute to an unfavorable vaginal environment in infertile women, potentially affecting sperm viability. The presence of *Oscillospira* spp. in fertile women suggests it is associated with a healthy vaginal microbiota profile.

## 1. Introduction

Infertility has emerged as a significant public health concern in developing nations, placing a substantial burden on healthcare and economies [1,2]. Globally, infertility affects 15–25% of couples during their reproductive years [3,4], with female infertility accounting for 37% of cases [5]. Female infertility has mainly been attributed to several genetic and pathophysiological mechanisms [3,6,7,8,9]. However, the factors responsible for 15–40% [6] of the cases of infertility in females remain unclear; these cases are often termed “idiopathic infertility” or “unexplained infertility” [7,10,11]. As such, clinicians perpetually endeavor to uncover the causes of these afflictions and, hopefully, increase fertility.

In recent decades, several studies have suggested that dysbiosis of the vaginal microbiota, characterized by cessation of *Lactobacillus* spp. dominance [12], may have several consequences, including infertility [10,13,14,15,16,17]. The vaginal microbiota can be classified into five community state types (CSTs I–V) [12]. In this field, the roles of CST I, CST II, CST III, and CST V [12,18,19,20,21] have received considerable attention, with some studies reporting greater *Lactobacillus* spp. abundance in fertile women. However, to date, there are no definitive conclusions regarding the specific microbe or optimal composition of vaginal microbiota in relation to female infertility. This inconsistency highlights the need to investigate less-studied CSTs, such as CST IV. Studies focusing on CST IV are limited, and existing evidence suggests that CST IV may contribute to the development of female infertility [12,21,22,23]. CST IV can be sub-grouped into CST IVA and CST IVB. CST IVA is characterized by the absence of *Lactobacillus* spp. but richness in *Prevotella* spp. and *Peptoniphilus* spp. CST IVB is characterized by low levels of *Lactobacillus* spp., with low proportions of various anaerobic bacterial microorganisms and an abundance of *Atopobium* spp., *Corynebacterium* spp., *Finegoldia* spp., and *Gardnerella* spp. [23,24,25]. The association between specific microorganisms of CST IVA or CST IVB in vaginal microbiota and infertility has not been investigated [26].

Thus, in this study, we aimed to analyze the differences in the composition of the vaginal microbiota, with a particular focus on CST IVA and CST IVB, between women with unexplained infertility and spontaneous conception followed by delivery.

## 2. Materials and Methods

### 2.1. Ethics Approval

This study was approved by the Research Ethics Committee of the Hualien Tzu Chi Hospital (IRB110-179-A). It was conducted in accordance with the Declaration of Helsinki and Good Clinical Practice Guidelines. All participants were provided with comprehensive details prior to taking part in this study. Each participant provided written consent to ensure their voluntary agreement to participate in the study.

### 2.2. Study Design and Subjects

We recruited 30 female participants from the Department of Obstetrics and Gynecology, Hualien Tzu Chi Hospital, from January 2022 to December 2022. This population was subdivided into two cohorts: a fertile cohort (*n* = 7), whose members had achieved previous spontaneous conception with full-term delivery, and an infertile cohort (*n* = 15), whose members had been diagnosed with infertility. Samples of fertile cases were collected from routine pap smear examinations. Samples of infertile cases were collected from the infertility clinic. The exclusion criteria included being under 20 years old, menopausal, or afflicted by symptomatic vaginitis, pelvic inflammatory disease, or structural anomalies of the uterus. Baseline characteristic data, including age and body mass index (BMI), were also collected.

### 2.3. Sample Collection, Bacterial DNA Extraction, and RNA Sequencing

Vaginal discharge was obtained using a cervical brush as part of a speculum examination. The specimens were preserved in DNA preservation liquid. The bacterial DNA from the vaginal discharge samples was extracted using the ZymoBIOMICS DNA Microprep kit (Zymo Research, Irvine, CA, USA) in accordance with the manufacturer’s instructions [27]. Polymerase chain reaction (PCR) amplification and library preparation of the variable regions (V3–V4) of the 16S rRNA gene were performed using the Quick-16STM next-generation sequencing (NGS) library prep kit (Zymo Research, USA). This library was constructed by incorporating unique identification indices for each sample through the use of the Nextera XT sequencing kit (Illumina, San Diego, CA, USA) to obtain and purify the V3–V4 amplicon. Then, the samples were diluted to a concentration of 4 nM for sequencing. The prepared library was quantified using a Qubit 2.0 Fluorometer (Thermo Fisher Scientific, Waltham, MA, USA) prior to sequencing, and the final library concentration was 8 pM. Library sequencing was performed using MiSeq Reagent Kit v3 (Illumina) reagents and MiSeq (Illumina) as the sequencing platform for the metagenomics workflow. Illumina local run manager software (version 3.0) was used for sequencing-data analysis. The index reads were demultiplexed, and FASTQ files were generated. The reads were classified against the Greengenes 16S rRNA gene database (version gg_13_5) [28], which achieved up to species-level sensitivity [29]. The results were generated using the default parameters in the original QIIME 2 software product (version 1.9.1).

### 2.4. Statistical Data Analysis

Continuous and categorical variables are presented as means ± standard deviations (SD) and frequencies or proportions, respectively. For comparisons of vaginal microbiota composition between the two groups, the relative abundance (percentage) of different bacterial taxa (at the genus or species level, when available) was calculated for each patient. Consequently, the Wilcoxon rank-sum test was adopted to compare the mean differences between the two groups for these taxa. Statistically significant differences were defined as *p* < 0.05. All statistical analyses were performed using the Statistical Package for the Social Sciences (SPSS) 21.0 (IBM Corp., Armonk, NY, USA).

## 3. Results

### 3.1. Flow Chart Pertaining to Study Design and Demographics

**Figure 1:**
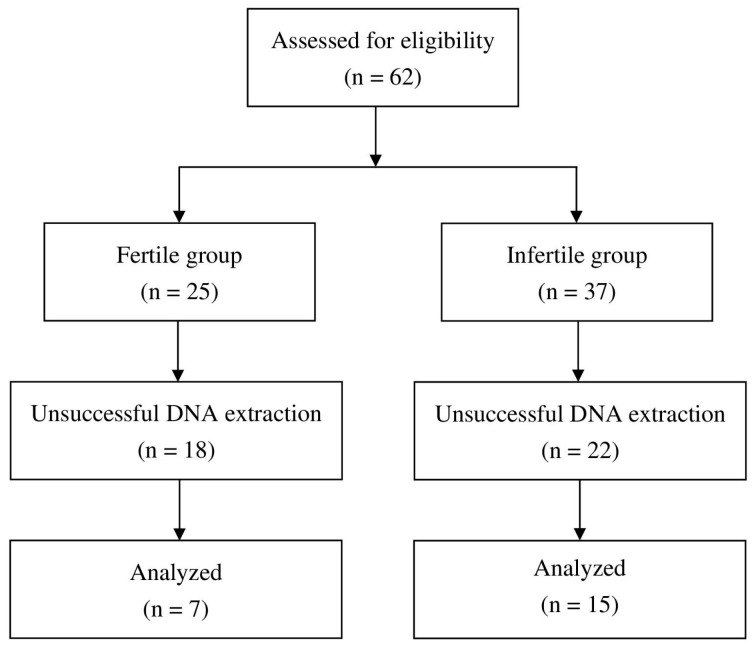
Flow chart of the study design.

Between January 2022 and December 2022, 37 infertile females and 25 fertile females were evaluated to determine their eligibility. Due to unsuccessful DNA extraction, 22 infertile and 18 fertile cases were excluded from further analysis. We ultimately included 22 participants, corresponding to 15 infertile cases and 7 fertile cases, in our investigation (Figure 1).

### 3.2. Vaginal Microbiota Distribution in the Cohort

**Figure 2:**
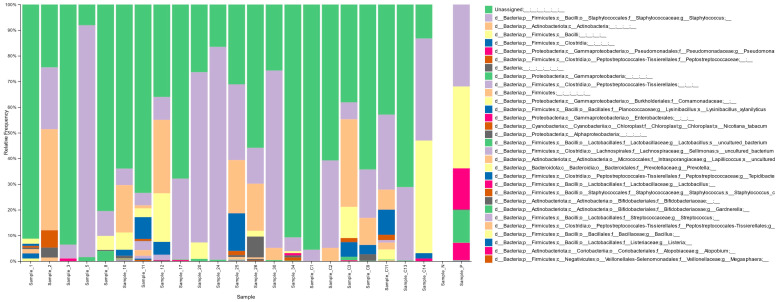
Stacked bar graph of the distribution of microbiota by bacterial taxa in infertile (Sample_1–34) and fertile females (Sample_C1–14), the negative control (Sample_N), and the positive control (Sample_P).

The stacked bar graph in Figure 2 shows the distribution of vaginal microbiota by bacterial taxa in infertile females (Sample_1–34), fertile females (Sample_C1–14), the negative control (Sample_N), and the positive control (Sample_P). The numbering system (Sample_1–34 and Sample_C1–14) included both successful and failed DNA extractions; only samples with sufficient sequencing quality were retained for analysis, resulting in 15 infertile samples and 7 fertile samples being included in the final dataset. The unclassified microbiota (depicted in green in Figure 2) was not considered in the analysis of microbiota composition.

### 3.3. Composition of Vaginal Microbiota in Fertile and Infertile Women

**Figure 3:**
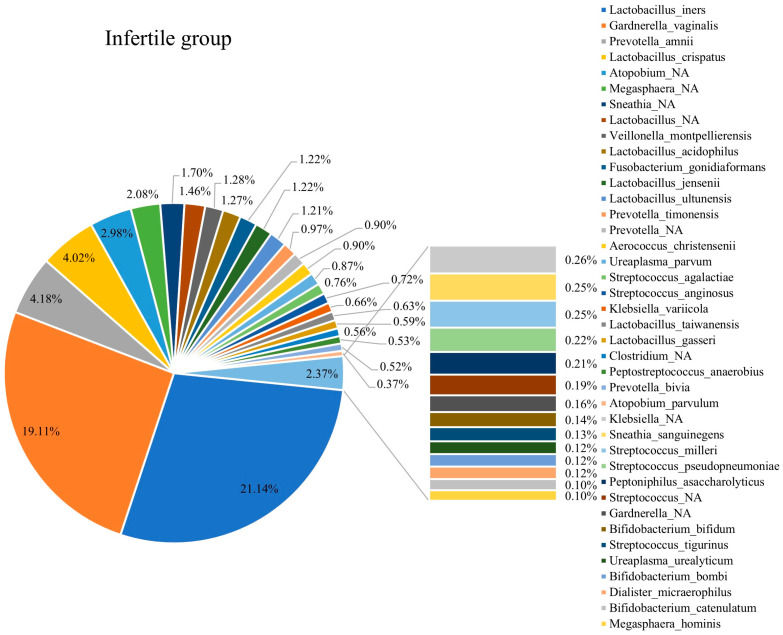
The composition of the vaginal microbiota in the infertile group.

**Figure 4:**
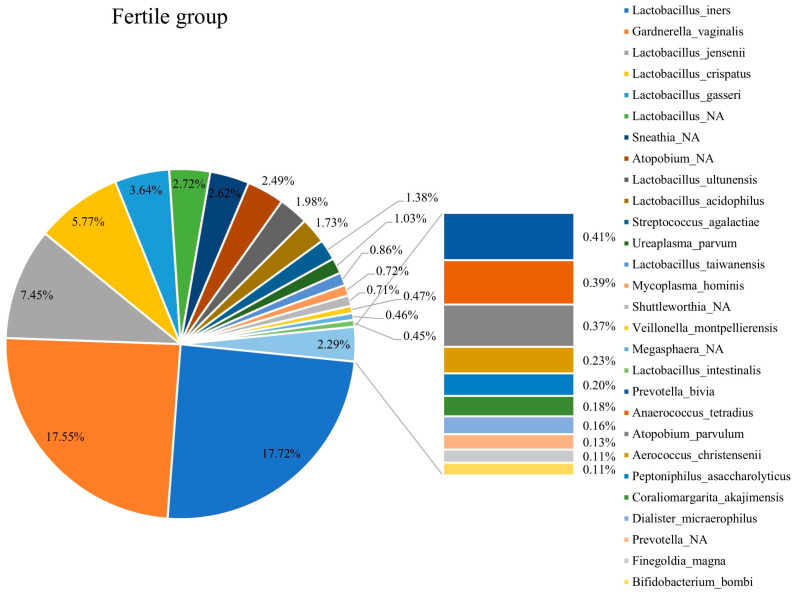
The composition of the vaginal microbiota in the fertile group.

Figure 3 and Figure 4 show the compositions of vaginal microbiota in infertile and fertile females. The top microbiota in infertile females included *L. iners*, *Gardnerella vaginalis*, *Prevotella amnii*, *L. crispatus*, and *Atopobium*. The top microbiota in fertile females included *L. iners*, *Gardnerella vaginalis*, *L. jensenii*, *L. crispatus*, and *L. gasseri*. The collective proportions of *Lactobacillus* spp. were 31.54% and 42.32% in the infertile and fertile females, respectively, reflecting a lower abundance of *Lactobacillus* spp. in infertile females.

### 3.4. CST IV Composition Between the Infertile and Fertile Groups

**Figure 5:**
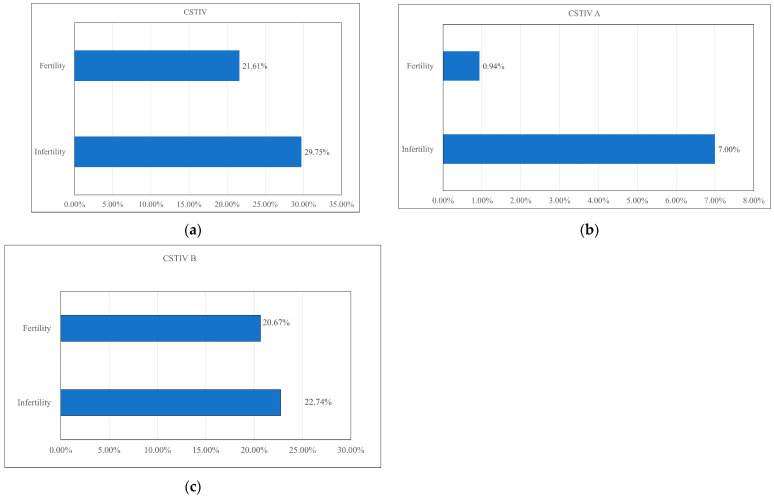
Percentage distribution of vaginal microbiota community state types (CSTs) between infertile and fertile women: (**a**) overall distribution of CST IV; (**b**) relative proportion of CST IVA; and (**c**) relative proportion of CST IVB. Data are presented descriptively without formal statistical testing. CST: community state type.

**Figure 6:**
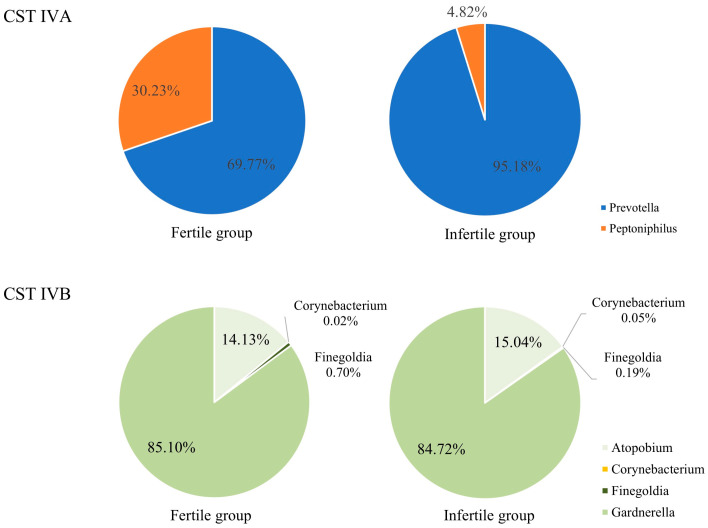
Percentage distribution of dominant bacterial genera in CST IVA (*Prevotella* and *Peptoniphilus*) and CST IVB (*Atopobium*, *Corynebacterium*, *Finegoldia*, and *Gardnerella*) among infertile and fertile women. Data are presented descriptively without formal statistical testing. CST: community state type.

Analyzing the differences in the abundance of CST IV bacteria in the vaginal microbiota between the two study groups revealed that infertile females (29.75%) had a higher value than fertile females (21.61%) (Figure 5a). Descriptive analysis revealed that CST IV was more frequent in infertile women (29.75%) than in fertile women (21.61%) (Figure 5a). CST IVA accounted for a higher proportion of the vaginal microbiota in infertile women (7.0%) in comparison with fertile women (0.94%) (Figure 5b), whereas CST IVB distributions appeared similar between the two groups (Figure 5c). At the genus level, *Prevotella* constituted a higher percentage of CST IVA in infertile women (95.18%) than in fertile women (69.77%) (Figure 6). These findings are based on percentage distributions, and no formal statistical testing was applied.

### 3.5. Comparison of Vaginal Microbiota Compositions in Infertile and Fertile Females

The compositions of the vaginal microbiota in infertile and fertile females, obtained using the mean value of each vaginal microbiota, are presented in Table 1. The data show that the amounts of *Kitasatospora_NA*, *Oscillospira_eae*, *Alkaliphilus_crotonatoxidans*, *L. farciminis*, *L. brantae*, *L. japonicus*, *Staphylococcus_aureus*, *and Corynebacterium_atypicum* were significantly higher in fertile females than in infertile females.

## 4. Discussion

This study resulted in four major findings. First, *Lactobacillus* spp. was more abundant in fertile patients than in infertile patients (Figure 3 and Figure 4). Second, CST IV appeared more frequently in infertile women (Figure 5a). Third, within CST IV, *Prevotella* spp. in CST IVA accounted for a larger proportion in infertile women relative to their fertile counterparts (Figure 6). Fourth, the relative abundance of bacterial taxa was significantly higher in fertile females than in infertile females (Table 1).

Burton et al. first employed the NGS method to characterize the vaginal microbiota in 2002. Since then, an increasing number of studies have focused on the relationship between vaginal microbiota and infertility, especially with respect to idiopathic recurrent pregnancy loss [30,31,32,33]. Most studies have established that the vaginal microbiota was dominated by *Lactobacillus* spp., classified into CSTs; among them, CST I, II, III, and IV are more abundant in fertile groups [7,12,13,20,28,34,35,36,37].

Although exploring the specific microorganisms of the vaginal microbiota that cause infertility has gained considerable attention, no conclusive findings have been reported [10,26,38]. Wee et al. enrolled 31 participants to compare vaginal microbiota compositions between infertile and fertile females. While no specific cluster showed differences between the two groups, a trend indicated that *Ureaplasma* and *Gardnerella* were more abundant in the vagina and cervix, respectively, of infertile females [26]. Sezer et al. also investigated the differences in vaginal microbiota between infertile and fertile females. They found that the amounts of *Lactobacillus* spp., *Staphylococcus* spp., *Streptococcus* spp., and members of the *Enterobacteriaceae* family were significantly higher in the infertile group under the age of 30. However, the relative abundance of *Gardnerella vaginalis*, *Eubacterium* spp., *Sneathia*, *Megasphaera* spp., *Peptostreptococcus* spp., *Atopobium vaginae*, and *Mycoplasma hominis* was significantly higher in the infertile group over the age of 30 [38]. Chopra et al. conducted a prospective study involving 80 participants and found that in infertile females, the amounts of *Gardnerella* spp., *Prevotella* spp., *Atopobium* spp., and *Enterococcus* spp. were significantly higher, whereas the proportions of *L. iners* were lower [10].

The vaginal microbiota is dynamic, and CST IV bacteria accumulate when *Lactobacillus* spp. are no longer dominant [35]. CST IVA is characterized by increased relative abundance or rates of *Peptoniphilus* spp. and *Prevotella* spp., and CST IVB is dominated by various anaerobic bacterial species related to bacterial vaginosis (BV), such as *Atopobium* spp., *Corynebacterium* spp., *Finegoldia* spp., and *Gardnerella* spp. [23,39,40,41,42]. Therefore, the presence of CST IVA may indicate that asymptomatic BV and CST IVB may develop into a clinical infection [42]. Previous studies have focused on the association between the presence of CST IV, especially CST IVB, and infertility [12,21,22,23,35]. To the best of our knowledge, no studies have explored the relationship between CST IVA and fertility in infertile and fertile females. In addition, CST IVA may cause asymptomatic BV, which may be a silent cause of female infertility. Furthermore, no studies have compared the differences between specific microorganisms in vaginal microbiota using subgroup analysis of CST IV between infertile and fertile females. We particularly focused on the specific microorganisms in CST IV that may be associated with infertility, given that CST IV plays an important role in infertility. In this study, subgroup analysis of CST IV showed that the most prevalent microorganisms were *Gardnerella vaginalis*, *Prevotella amnii*, and *Atopobium* spp. Among CST IVA compositions, the relative abundance of *Prevotella* spp. was higher in infertile females (95.18%) than in fertile females (69.77%). This finding suggests a possible association between CST IVA and asymptomatic BV in infertile women. Previous studies reported that *Prevotella* spp. may negatively affect semen quality, contributing to reduced motility and concentration of sperm [43,44,45,46,47], and correlations between vaginal and seminal microbiota in idiopathic infertile couples have been observed [48,49]. While these reports provide a biological rationale, we did not directly assess semen quality. Therefore, the interpretation that *Prevotella* spp. may impair sperm function should be regarded as hypothetical. Future studies are needed to directly evaluate semen–vaginal microbiome interactions to clarify this relationship.

We found that several bacterial microorganisms were prominent in fertile females, including *Kitasatospora* spp., *Oscillospira* spp., *Alkaliphilus crotonatoxidans*, *L. farciminis*, *L. brantae*, *L. japonicus*, *Staphylococcus aureus*, and *Corynebacterium atypicum*. Among them, *Oscillospira* is the most clinically important. Wu et al. developed an intrauterine mouse model and found that *Oscillospira* spp. serve as beneficial vaginal bacteria, increasing the abundance of *Lactobacillus* and restoring the balance of the vaginal microbiota [50]. *Oscillospira* spp. could be a promising candidate for next-generation probiotics, offering potential benefits for fertility.

We compared the differences in vaginal microorganisms between infertile and fertile females using CST IV subgroup analysis. Our findings may fill in the existing knowledge gap. Nevertheless, our study still suffers from some limitations. First, the final sample was relatively small (*n* = 22), reducing statistical power and potentially limiting the generalizability of the findings. Moreover, more than 60% of the original samples did not make it through DNA extraction, and this failure may have introduced selection bias. The potential causes of extraction failure include low microbial biomass, technical variability in specimen handling, or storage-related DNA degradation. Also, there are no standardized methods for DNA extraction for vaginal microbiota [51]. Second, as only participants from a single center in Taiwan were enrolled, the results may not be generalizable to other populations. Third, we only reported relative abundances and percentage differences without providing exact *p*-values or effect sizes, restricting the statistical transparency of our findings. Another limitation of this study is that information on potential confounding factors such as recent antibiotic use, sexual practices, hormonal treatments, or lifestyle variables was not collected. These factors are known to substantially influence vaginal microbiota composition and could have impacted our findings. Future studies should incorporate detailed records of these variables to better control for their effects. Finally, taxonomic classification relied on the Greengenes database, which is incomplete for vaginal-specific bacteria, leaving some organisms unclassified. These limitations should be addressed in future large-scale, multicenter, and methodologically standardized studies.

## 5. Conclusions

This study demonstrated that infertile women exhibited a higher prevalence of CST IV, particularly CST IVA, than fertile women. Within CST IVA, the relative abundance of *Prevotella* spp. was markedly elevated in infertile subjects, suggesting a potential association with an unfavorable vaginal environment. Conversely, *Oscillospira* spp. were more abundant in fertile women, which may indicate a healthy vaginal microbiota. While previous reports have linked *Prevotella* spp. to impaired semen parameters, our study did not directly assess semen quality; therefore, such interpretations remain hypothetical. Future large-scale, multicenter studies incorporating integrated analyses of vaginal and seminal microbiota are required in order to validate these findings and explore the potential clinical applications of vaginal microbiota in infertility diagnosis and treatment.

## Figures and Tables

**Table 1:** The compositions of the vaginal microbiota in infertile and fertile females, presenting the mean values for each vaginal microbiota.

Bacterial Taxa	Infertility (*n* = 15)		Fertility (*n* = 7)		Total		*p*-Value
	Mean	SD	Mean	SD	Mean	SD	
Kitasatospora_NA	0.000	0.000	0.078	0.081	0.025	0.057	0.001
Oscillospira_eae	0.004	0.017	0.041	0.055	0.016	0.037	0.025
Alkaliphilus_crotonatoxidans	0.000	0.000	0.018	0.031	0.005	0.019	0.030
Lactobacillus_farciminis	0.000	0.000	0.034	0.059	0.011	0.035	0.030
Lactobacillus_brantae	0.008	0.033	0.125	0.201	0.011	0.035	0.037
Lactobacillus_Japonicus	0.000	0.000	0.025	0.046	0.008	0.027	0.039
Staphylococcus_aureus	0.010	0.027	0.064	0.091	0.027	0.059	0.044
Corynebacterium_atypicum	0.000	0.000	0.085	0.158	0.027	0.094	0.045

Data are presented as rates per ten thousand (‱).

## Data Availability

All data generated or analyzed during this study are available in publicly accessible repositories. The 16S rRNA gene sequencing data used to analyze the vaginal microbiota in this study have been deposited in the DNA Data Bank of Japan (DDBJ) under BioProject accession number PRJDB18901, with sample accession numbers SAMD00824136-SAMD00824157.

## Abbreviations

The following abbreviations are used in this manuscript:

CST	Community state type
BMI	Body mass index
PCR	Polymerase chain reaction
NGS	Next-generation sequencing
SD	Standard deviation
BV	Bacterial vaginosis
